# Effects of probiotic supplementation on faecal short-chain fatty acid concentrations in healthy individuals: a systematic review and meta-analysis of randomized controlled trials

**DOI:** 10.1186/s12937-026-01364-0

**Published:** 2026-07-13

**Authors:** Kincső Csepke Földvári-Nagy, Yasmin Zubeida Al-Gharati, Dániel Sándor Veres, Tamás Schnabel, Katalin Lenti, Anna Júlia Éliás, László Földvári-Nagy

**Affiliations:** 1 Faculty of Health Sciences, Semmelweis University, Budapest, 1088 Hungary; 2 School of Life Sciences, University of Warwick, Coventry, CV4 7AL UK; 3 Department of Biophysics and Radiation Biology, Semmelweis University, Budapest, 1094 Hungary; 4 Department of Gastroenterology, Skien Hospital, Telemark Hospital Trust, Skien, 3710 Norway; 5 Department of Morphology and Physiology, Faculty of Health Sciences, Semmelweis University, Budapest, 1088 Hungary

**Keywords:** Probiotics, Butyrate, Acetate, Propionate, Short-Chain Fatty Acids, SCFA

## Abstract

**Background:**

Short-chain fatty acids produced by microorganisms living in the gastrointestinal tract are known to have numerous beneficial effects. Because certain probiotic strains may influence short-chain fatty acid production, probiotic supplementation has been proposed as a potential strategy to modify faecal short-chain fatty acid concentrations. Our aim was to investigate whether probiotics modify faecal short-chain fatty acid levels in healthy populations via systematic review and meta-analysis.

**Methods:**

We performed a systematic search of the MEDLINE, Embase, and Cochrane databases on 12/04/2024. We analysed exclusively randomized controlled trials meeting all the following criteria: population: healthy people; intervention: probiotic supplementation; control: no probiotic supplementation; outcome: faecal short-chain fatty acid concentrations. A total of 9217 articles were reviewed, 21 of which met the preset inclusion criteria. Twelve articles, including 541 subjects, were eligible for meta-analysis of changes in faecal short-chain fatty acid concentrations. The standardized mean difference (SMD, Hedges’ g) was chosen as the effect indicator due to the different measurement techniques used. A random-effects model was used to estimate SMD with 95% confidence interval (CI) due to the expected heterogeneity.

**Results:**

No statistically significant differences were detected in the faecal butyrate (SMD = 0.07, 95%CI:[(-)0.16 – 0.30]), acetate (SMD = 0.06, 95%CI:[(-)0.14 – 0.27]), and propionate (SMD = 0.09, 95%CI:[(-)0.07 – 0.25]) levels between individuals taking and not taking probiotics.

**Conclusions:**

Our results suggest that probiotic intake has a minor effect on faecal short-chain fatty acid concentrations, but this effect is not statistically detectable in healthy individuals. Thus, there is insufficient evidence of statistically significant effect of probiotic supplementation on stool short-chain fatty acid concentrations in healthy individuals.

**Trial registration:**

Research protocol was registered in PROSPERO (CRD42022286137).

**Supplementary Information:**

The online version contains supplementary material available at https://doi.org/10.1186/s12937-026-01364-0.

## Introduction

Probiotic products contain live microorganisms that, when administered in adequate amounts, confer a health benefit on the host [1]. Probiotics generally consist of microorganisms present in the native gut microbiota, including bacteria, yeasts, or a combination thereof [2]. These products commonly contain strains from bacterial genera such as *Lactobacillus*, *Bifidobacterium*, and *Bacillus*. Probiotics are widely perceived as products that support gut and digestive health, and many consumers believe that they may help restore the balance and diversity of the gut microbiota, particularly after antibiotic use or dietary changes [3, 4]. These products are also partly offered to healthy people as a general preventative approach, largely because of increased public knowledge of the alleged health advantages of probiotics, including enhanced digestive system performance and gut health [3]. While previously mostly functional fermented milk products such as yogurt were in focus, the market is now diversifying into other probiotic foods, drinks, and supplements, including fruit- and vegetable-based products, cereal-based products, and other non-dairy probiotic matrices [5, 6]. Reflecting this growing demand, the estimated value of the worldwide probiotics market was USD 77.12 billion in 2022 [3], with an anticipated compound annual growth rate of 14.0% projected for the period between 2023 and 2030 [3].

Recently, SCFAs have received much attention in the context of gut microbiome studies. These SCFAs include acetate, propionate, and butyrate. These SCFAs are produced by the gut microbiota through the fermentation of dietary fibres in the intestinal lumen [7]. However, probiotic effects on SCFA production are likely to be strain-specific and may depend on interactions with the resident gut microbiota and the availability of fermentable substrates [8]. Notably, several commensal gut genera, including *Faecalibacterium* and *Roseburia*, are recognized as important butyrate producers [9].

SCFAs have several beneficial effects by contributing to cellular energy metabolism, epithelial barrier maintenance, and immune regulation, thereby supporting gut homeostasis. SCFAs have been shown to exert therapeutic and preventive effects in a wide range of diseases, including obesity, cardiovascular disease, liver disease, diabetes mellitus, inflammatory bowel diseases, diarrhoea, constipation, neurodegenerative diseases, neuropsychiatric diseases, cancers, arthritis, and periodontal disease as summarized in a recent review [10]. Furthermore, a variety of underlying processes are involved, with particular emphasis placed on the Nrf2, HDAC, NF-κB, and GPR41 signalling pathways [10].

Most of the available evidence on the biological and health-related effects of SCFAs derives from studies on butyrate, which is primarily considered to have an immune modulating [11–15], metabolic regulating [16–18], cardiovascular disease-preventing effect [19, 20], and a role in colon cancer prevention [21–23], among other functions. While previous studies focused primarily on butyrate, the effects of other SCFAs are increasingly becoming a focus of interest. For example, acetate availability, metabolism, and signalling have all been connected in different ways to diabetes, obesity, heart diseases, cancers, and liver diseases [24–26]. According to several studies, propionic acid has antilipogenic and cholesterol-lowering effects while controlling weight and feeding behaviour [27, 28]. Furthermore, similar to butyrate, propionate has a protective effect against colon cancer [28, 29]. In comparison to healthy patients, those at risk of developing colorectal cancer (CRC) had considerably lower concentrations of the three major faecal SCFAs, according to a recent systematic review and meta-analysis [30]. Altered faecal butyrate levels have been reported in patients with colorectal cancer, suggesting a potential association with disease characteristics [31]. Based on these observations, one possible way to improve CRC prevention and clinical outcomes is to modify the gut microbiota composition towards species that are more conducive to the production of SCFAs. However, more human studies are still needed, as much of the available evidence currently derives from animal or preclinical research [10].

Although most SCFAs produced in the colon are absorbed and only a small fraction is excreted in the faeces [32], faecal SCFA analysis remains a practical and non-invasive approach for assessing colonic microbial fermentation in human studies [7, 33]. Accordingly, we focused on faecal SCFA concentrations as the most feasible outcome for synthesis across randomized controlled trials.

While SCFA production is considered a widespread property among commonly studied probiotics [1], the effects of probiotic supplementation on measurable faecal SCFA concentrations in the general healthy population have not been summarized to our knowledge. Therefore, we conducted a systematic review and meta-analysis to assess the effect of probiotic supplementation on faecal SCFA concentrations in healthy individuals, presenting here a focused analysis from our broader systematic review.

## Methods

We structured our study in accordance with the Cochrane recommendations [34]. To present our findings, we adhered to the guidelines outlined in the Preferred Reporting Items for Systematic Reviews and Meta-Analyses (PRISMA) 2020 Statement [35] (Supplementary Material 1: Table S1). Our study protocol was previously submitted to the International Prospective Register of Systematic Reviews (PROSPERO) (CRD42022286137).

### Systematic search and selection

We formulated our clinical question and set the eligibility criteria using the PICO-S format, which encompasses population, intervention, comparison, outcome, and study design. All studies meeting the following criteria were included in our analysis: *population (P)* — healthy people (as stated in each manuscript); *intervention (I)* — probiotic supplementation (in the form of a supplement, e.g., capsule or powder); *comparison group (C)* — no probiotic supplementation (placebo or no supplementation). The assessed *outcomes (O)* — *primary outcome*: in the broader review included gut microbial diversity (any diversity indices reported) at the end of the intervention and after a follow-up period, as reported in each study; *secondary outcome*: gut microbiome composition at the end of the intervention and after a follow-up period, as reported in each study; *additional outcomes*: any other clinical parameters reported in the included studies, if sufficient data were available (including faecal SCFA concentrations). The present publication specifically focuses on faecal SCFA concentrations in healthy individuals. We did not apply restrictions on sex, age, or ethnicity. We included only randomized controlled trials (RCTs).

We conducted a systematic search across three medical databases – MEDLINE (via PubMed), Embase (via embase.com), and the Cochrane Central Register of Controlled Trials (CENTRAL) – without applying filters or restrictions. The search utilized the key provided in Supplementary Material 1: Table S2 and was carried out until 12/04/2024. The search key was designed to find all RCTs that investigated the effects of probiotics in healthy people.

In cases where our search did not yield published protocols for the identified eligible studies, we attempted to find them on the EU Clinical Trials Register website [36] and on the ClinicalTrials.gov website [37].

The selection was performed with the reference management program EndNote X9 (Clarivate Analytics, Philadelphia, PA, USA). Following the automated and manual removal of duplicates process was performed stepwise. Because of the high number of records remaining after duplicate removal, both the title/abstract screening and the full-text screening were conducted by two independent reviewer pairs, with each pair assessing approximately half of the records. Cohen’s kappa coefficient was calculated for each reviewer pair at each selection stage to describe inter-reviewer agreement. Disagreements were resolved through consensus. In case of meeting the general inclusion criteria, studies were divided into subgroups and analysed according to specific outcomes. The present paper summarizes the results of the subgroup of studies investigating the effect of probiotics on faecal SCFA concentrations.

### Data collection

In the data extraction process, two independent authors manually gathered information from each article, crosschecking each other's data pool. Any discrepancies were resolved through consensus. The collected information was then summarized via a standardized data collection form. The extracted data included the following: study characteristics (first author, year of publication, country, number of centres, and setting), study sample description (sample size, sex distribution, age, and any specificity of the sample as stated in each study), details for probiotics (probiotic type, dose, and duration) and outcomes as reported in each article. In instances where data were presented solely in graphical format, we utilized GetData Graph Digitizer software (version 2.26.0.20) to extract the information [38].

### Synthesis methods

A biostatistician conducted the statistical analysis using the *R* software (v4.3.2) [39] with the *meta* (v6.5.0) [40] and *dmetar* (v0.1.0) [41] packages. Different studies have reported results at different scales, using various measurement methods (e.g., concentrations calculated on dry or wet faeces) and various units of measurement. Therefore, standardized mean differences (SMDs, bias corrected exact Hedges’ g [42]; probiotic minus control) with 95% confidence intervals (CIs) for the effect size measure were calculated. When the means and corresponding standard deviations (SDs) were available, they were directly extracted. In cases where this information was not provided, but quartiles (extracted from box plots) were available, the Luo [43] and Shi [44] methods were used as implemented in the *meta* package to estimate the mean and SD. On the basis of the article of Gargari et al. [45], where the individual raw data of butyrate, acetate, and propionate levels were given, we could assume that the distribution of these faecal concentrations did not differ from a normal distribution in relevant amounts. Therefore, the estimation of the mean and SD from the quantiles could be acceptable. We pooled the SMD values of the faecal concentrations of butyrate, acetate, and propionate after treatment and used the inverse variance weighting method for each of them separately. As we exclusively included RCTs, it was assumed that the characteristics before treatment were not different between the intervention and control groups. A sensitivity analysis was conducted for data before the intervention to reveal any baseline differences that may be present. Furthermore, as some of the studies used a crossover design but reported no data for the first intervention period, and we could not analyse them as a parallel study, we performed a subgroup analysis based on the study design. Additionally, we synthesized our data, forming subgroups according to the level of risk of bias and the composition of the probiotic supplementation in each study. For the probiotic-composition subgroup analyses, studies were grouped according to the composition of the probiotic formulation used in each trial (e.g., *Bacillaceae*-containing, *Bifidobacteriaceae*-containing, *Lactobacillaceae*-containing, mixed *Lactobacillaceae/Bifidobacteriaceae* formulations, or other mixed lactic acid bacteria formulations). Given the anticipated between-study heterogeneity, a random-effects model was employed for effect size pooling. Hartung-Knapp adjustment was applied for the CI of the pooled effect [46, 47]. We applied a maximum-likelihood estimator with the Q profile method for confidence intervals to estimate the heterogeneity variance measure τ^2^ [48]. Between-study heterogeneity was also described using Higgins & Thompson’s I^2^ statistics [49]. Publication bias was assessed via visual inspection of funnel plots. Potential influential studies were explored using leave-one-out analyses.

The results were presented via forest plots illustrating the SMD with corresponding 95% CI lower and upper limits for individual studies. We also reported the prediction interval when at least 10 studies were available and between-study heterogeneity was not considerable (defined as I^2^ < 75%). The confidence intervals for individual studies were depicted on the plot using t-distribution estimation. The findings are reported as (SMD [95% CI lower limit – 95% CI upper limit]). In case of subgroup (risk of bias level, cross-over study design, composition of the probiotic supplementation) analysis, we used a fixed-effects “plural” model (aka. mixed-effects model). We assumed that all subgroups share a different τ^2^ value as we anticipate differences in the between-study heterogeneity, and the study number is not too small in subgroups. If at least one of the subgroups contains less than 6 studies, we use an assumption of the same τ^2^ at subgroups (recommended in Harrer et al. [50]).

### Risk of bias assessment

Two independent authors performed a risk-of-bias assessment using the revised Cochrane risk-of-bias tool (RoB2) [51]. Any disagreements were resolved through consensus. The domains assessed biases arising from the randomization process, deviations from the intended intervention, missing data, the measurement of the outcome, and the selection of the reported results. Investigators assigned a rating to each domain, and the algorithm automatically determined the risk level, categorized as low, some concerns, or high.

### Certainty assessment

Two independent investigators applied the Grading of Recommendations, Assessment, Development, and Evaluation (GRADE) tool to evaluate the quality of the best available evidence [52]. Any disagreements were resolved through consensus.

## Results

### Study selection

**Fig. 1:**
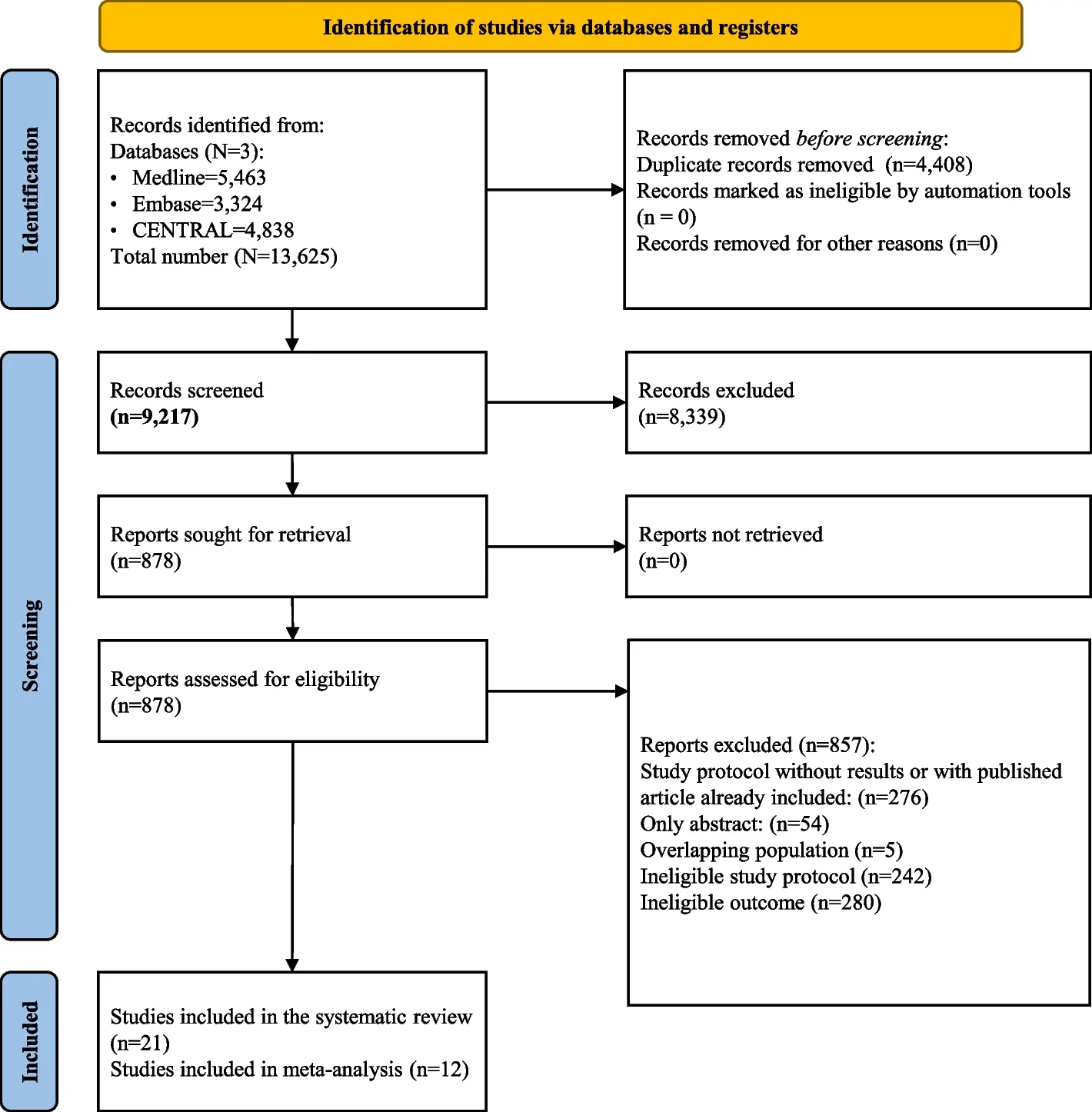
PRISMA flowchart of the selection process

The outcomes of the search and selection procedures are summarized in Fig. 1. Our search key initially pinpointed 13,625 records. The Cohen’s kappa indices for the two reviewer pairs in the title and abstract selection phase were 0.94 and 0.81, respectively, whereas those for two reviewer pairs in the full-text selection were 0.93 and 0.95, respectively. In total, 21 articles met the eligibility criteria for the qualitative synthesis of faecal SCFA concentrations. Our review exclusively included non-overlapping study populations based on the available information. Eligible studies focused predominantly on adult populations [45, 53–69], of which two investigated elderly individuals [53, 61]. Three articles specifically examined infants [70–72]. All studies were randomized and placebo-controlled; six used a crossover design [45, 58–62].

Although we identified twenty-one eligible articles reporting the results of faecal SCFA concentrations [45, 53–63, 65–73], only twelve for butyrate [45, 53, 55, 56, 58–61, 63, 64, 66, 68] and ten for acetate [45, 53, 55, 56, 59–61, 63, 64, 66] and eleven for propionate [45, 53, 55, 56, 59–61, 63, 64, 66, 68] contributed data, either in acceptable numerical format or via boxplots, for incorporation into the meta-analysis. Six articles reported the results in a way that we could not use for our quantitative synthesis, e.g., providing only the change values [62] or least-square-mean changes [22], not specifying the measurement levels on the figures [54, 70], not correctly readable data regarding acetate and propionate [58], or reporting confusing data in the tables when calculating back the baseline values from the after results [65]. Furthermore, Nakamura et al. organized results based on responder scores [67]. We did not include articles reporting data on the infant population. Since we had only two of these studies, we did not perform subgroup analysis on them either [71, 72]. We excluded the above articles from the meta-analysis and included them only in the systematic review to preserve the reliability of our results.

### Study characteristics

The main characteristics of the included studies can be found in Table 1.

**Table 1:** The main characteristics of the included studies

**Study**	**Country**	Study design (No. of centres*)	**Population (Randomized)**								
			**Number of randomized subjects** **(female %)**	**Number of subjects in probiotic group**	**Number of subjects in control group**	**Age (mean ± SD in years)** **in the intervention (and control) groups**	**Specification of the population**	**Probiotic type and dose**	**Placebo type and dose**	**Duration**	**Wash-out period (days) if applicable**
Alba (2024) [72]	Spain	randomized, double-blind, controlled trial (2)	187 (only available for compliant participants: 43)	90	97	*weeks* ± *SD* 12.1 ± 0.7 (12.1 ± 0.6) (compliant population)	Healthy, full-term, three-month-old infants, exclusively formula-fed	control formula plus *Bifidobacterium breve* DSM32583 at a concentration dose of 1 × 10^7^ CFU/g of powdered formula	commercial infant formula (HiPP Biológico, Leche de Inicio HiPP 1, 600 g)	3 months	N.A.
Bazanella (2017) [70]	Germany	double-blind, randomized, and placebo-controlled clinical trial	106 in 3 groups, of which 97 were eligible in two groups (64)	48	49	newborns (newborns)	healthy infants	*Bifidobacterium bifidum BF3,* *B. breve BR3, Bifidobacterium longum subspecies* *infantis BT1,* and *Bifidobacterium longum BG7* a total concentration of 10⁸ CFU/g	whey based infant formula	1 year	N.A.
Boesmans (2018) [59]	Belgium	randomized, double-blind, placebo-controlled crossover trial	30 (53)	15 (probiotic first)	15 (probiotic first)	mean (range) 32 (26–45) (28 (25–33))	healthy volunteers	*Butyricicoccus pullicaecorum 25-3 T* 10⁸ CFU/day	maltodextrin	28 days	21 days
Chaiyasut (2022) [53]	Thailand	randomized, double-blind, placebo-controlled trial	48 (79)	24	24	61.63 ± 0.84 (58.79 ± 1.21)	healthy elderly subjects	*Lactobacillus paracasei HII01* (2 × 10^1^⁰ CFU/day*); Bifidobacterium breve* (2 × 10^1^⁰ CFU/day); *Bifidobacterium longum* (10^1^⁰ CFU/day)	10 g corn starch	84 days	N.A.
Childs (2014) [62]	UK	double-blind, placebo-controlled, randomized, factorial crossover study	44 (50)	N.D.	N.D.	43 ± 12 (all participants)	healthy volunteers	*Bifidobacterium animalis subsp. lactis Bi-07 ATCC SD5220* 10⁹ CFU/day	maltodextrin	21 days	28 days
Ferrario (2014) [58]	Italy	randomized, double-blind, crossover placebo-controlled study	30 (60)	30	30	35 ± 10.7	healthy volunteers	*Lactobacillus paracasei DG* at least 2.4 × 10^1^⁰ viable cells	placebo	28 days	28 days
Freedman (2021) [56]	USA	parallel arm, double-blind, randomized, placebo-controlled intervention study	93 in 4 groups, from which 46 were eligible in two groups (61)	25	21	36.9 ± 12.9 (34.4 ± 13.0)	normal weight to mildly obese healthy volunteers	*Bacillus subtilis strain DE111* 10⁹ spores CFU/day	maltodextrin	28 days	N.A.
Gargari (2016) [45]	Italy	randomized, double-blind, crossover, and placebo-controlled intervention study	35	35	35	NI for the randomized population	healthy volunteers	*Bifidobacterium bifidum Bb* 3.8 × 10⁹ CFU/day	maltodextrin	28 days	28 days
Hiraku (2023) [71]	Japan	placebo-controlled, double-blinded randomized trial	111 (only available for compliant participants: 52)	57	54	newborns (newborns)	healthy full-term infants	*Bifidobacterium infantis* M-63 (1 billion CFU/1.0 g of sachet)	sterilized dextrin only/1.0 g of sachet	3 months	N.A.
Maneerat (2013) [61]	UK	randomized, double-blind, placebo-controlled, cross-over human clinical trial, but only the first part was considered to be valid	40 in four groups, from which 20 were eligible in two groups (available for the reduced number or participants (47))	10	10	available for the reduced number of participants (mean ± SEM) 64.6 ± 0.5 (63.0 ± 0.5)	good general health elderly volunteers (> 60 years)	*Bifidobacterium lactis Bi-07* 10⁹ CFU/day	maltodextrin (8 g/day)	21 days	28 days (N.A.)
Nakamura (2022) [67]	Japan	randomized, double-blind, controlled crossover trial	24 (60)	12	12	47.7 ± 5.8 (all participants)	healthy volunteers with constipation	*Bifidobacterium longum* BB536 5.0 × 10^9^ CFU/day	potato starch	14 days	28 days
Nyangale (2015) [60]	United Kingdom	triple-blind, placebo-controlled crossover design	42 (40)	N.D.	N.D.	65–80 (all participants)	healthy volunteers	*Bacillus coagulans GBI-30, 6086* 10⁹ CFU/day	microcrystalline cellulose	28 days	21 days
Pagliai (2023) [69]	Italy	randomized, double-blinded parallel controlled trial	40 (50)	20	20	51 ± 12.7 (51 ± 14.7)	overweight or obese (body mass index (BMI) 28.0–34.9) but otherwise overweight population (BMI ≥ 25 kg/m2)	*Lactiplantibacillus plantarum* IMC 510® 15 billion live cells CFU/capsule	placebo	3 months	N.A.
Park (2020) [57]	Korea	double-blind, randomized, placebo-controlled	70 (66)	35	35	48.3 ± 11.6 (8.3 ± 13.2)	healthy volunteers	*Lactobacillus plantarum LPQ180* 2 × 400 mg/2 × 4x 10⁹ CFU/day	2 × 400 mg maltodextrin	84 days	N.A.
Rahayu (2021) [55]	Indonesia	randomized, double-blind, placebo controlled study	60 (60)	30	30	44.07 ± 6.23 (44.67 ± 5.66)	healthy overweight volunteers (BMI > = 25)	*Lactobacillus* *plantarum Dad-13* 2 × 10⁹ CFU/day	1 g skimmed milk powder	90 days	N.A.
Sato (2022) [68]	Japan	double-blind, placebo-controlled, parallel-group comparison study	50 (43)	30	30	46.3 ± 8.0 (47.4 ± 11.5)	healthy Japanese adults who routinely lived under stressful conditions	*Pediococcus acidilactici* KABP-021 (CECT7483), *Lactiplantibacillus plantarum* KABP-022 (CECT7484), *and L. plantarum* KABP-023 (CECT7485*)* at the concentration of 1 × 10^9^ CFU/strain	starch	28 days	N.A.
Senda-Sugimoto (2024) [66]	Japan	randomized, double-blind, placebo-controlled, parallel-group trial	110 (51)	55	55	40.9 ± 11.5 (40.8 ± 11.2)	healthy volunteers	placebo + *Lacticaseibacillus paracasei* 327 25 mg (approximately 5 × 10^10^ CFU/day)	Glucose, starch, calcium stearate, fine granular silica, hydroxypropyl cellulose	14 days	N.A.
Sierra (2010) [63]	Spain	randomized, double-blinded, placebo- controlled study	40 (50)	20	20	33.0 ± 7.9 (all participants)	healthy volunteers	*Lactobacillus salivarius* CECT5713 2 × 10⁸ CFU/day	maltodextrin	28 days	N.A.
Son (2020) [54]	Korea	randomized controlled trial	20 (0)	10	10	only available for the participants without the drop-outs 26.50 ± 5.01 (27.14 ± 5.93)	bodybuilders who consumed an extremely high-protein/low-carbohydrate diet	*Lactobacillus acidophilus,* *L. casei, L. helveticus, and Bifidobacterium bifidum* 10^12^ CFU of each/day	corn starch	60 days	N.A.
Stenman (2016) [64]	Finland	double-blind, randomized, placebo-controlled, multicentre clinical trial (4)	225 in 4 groups 112 in the eligible two groups (available for the ITT population) (80)	55	57	available for the ITT population (48 and 56 subjects) 50.6 ± 10.6 (49.9 ± 8.5)	overweight or obese (body mass index (BMI) 28.0–34.9) but otherwise healthy volunteers	*Bifidobacterium animalis subsp.* *lactis 420*™ (B420), 10^1^⁰ CFU/day	12 g/day of microcrystalline cellulose	6 months	N.A.
Swanson (2002) [65]	USA	randomized, double-blind, placebo-controlled, parallel study	68 (only available for compliant participants: 62 in four groups 30 in the eligible two groups (57))	15 (compliant)	15 (compliant)	available for the compliant participants 27.1 ± 6.7 (25.1 ± 4.0)	healthy adult volunteers	*Lactobacillus acidophilus (LAC)* 10⁹ CFU/day	80 mg cornstarch	28 days	N.A.

*Abbreviations*: *USA* United States of America, *UK* United Kingdom, *N.D.* no data, *N.A.* not applicable, *CFU* Colony Forming Unit, *ITT* intention-to-treat

^*^If not otherwise mentioned, the studies were single centres

### The impact of probiotic supplementation on the faecal butyrate levels in healthy individuals

**Fig. 2:**
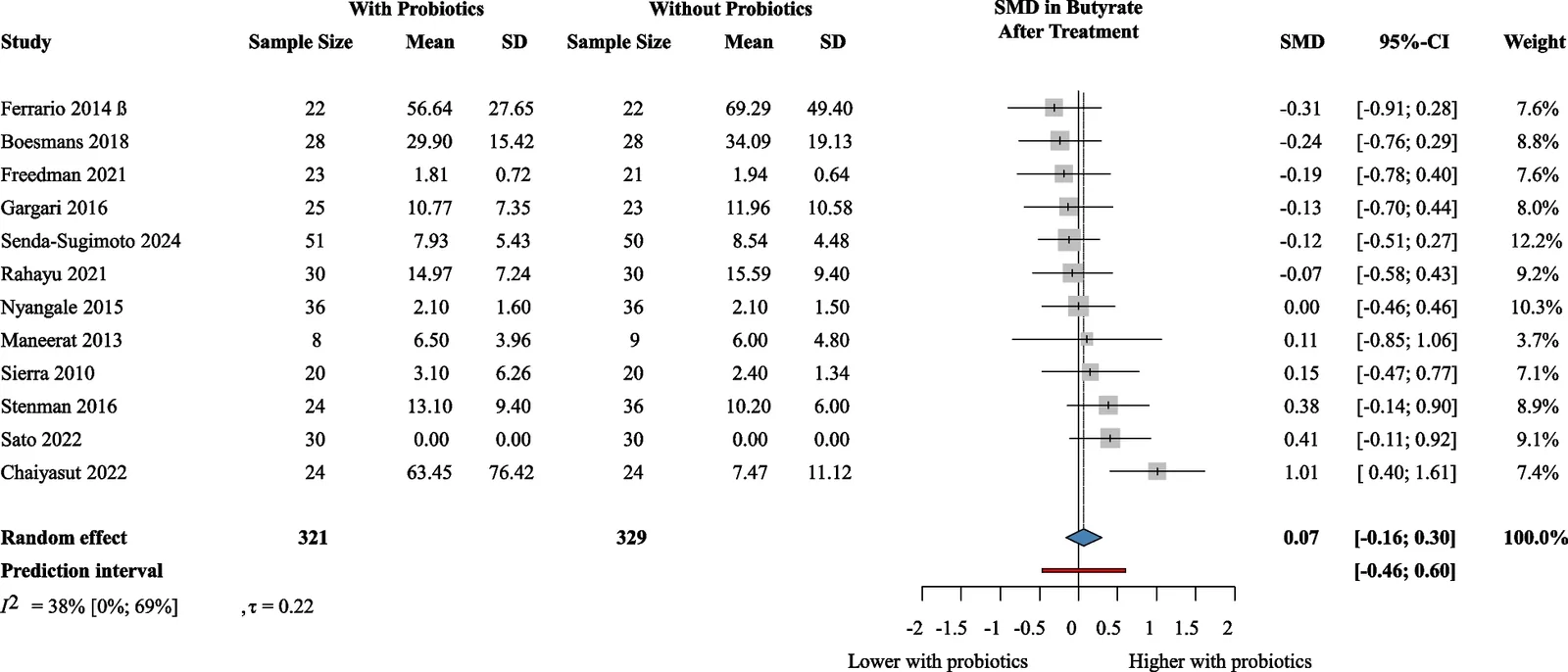
Forest plot of butyrate concentration results. The faecal butyrate concentration was not significantly higher in healthy people receiving probiotic supplementation than in those in the control group, as measured immediately after the treatment period. CI: confidence interval; SD: standard deviation; SMD: standardized mean difference. “ß” indicates that the mean and SD are estimated from quantiles in that study

The results of the meta-analysis of butyrate concentration, which included twelve articles with 541 participants [45, 53, 55, 56, 58–61, 63, 64, 66, 68], are summarized in Fig. 2. On the basis of our results, the faecal butyrate level was not statistically significantly different between the probiotic-supplemented and control groups when it was measured immediately at the end of treatment. The standardized mean difference in the faecal butyrate concentrations between the intervention and control groups was 0.07 [(-)0.16–0.30].

**Fig. 3:**
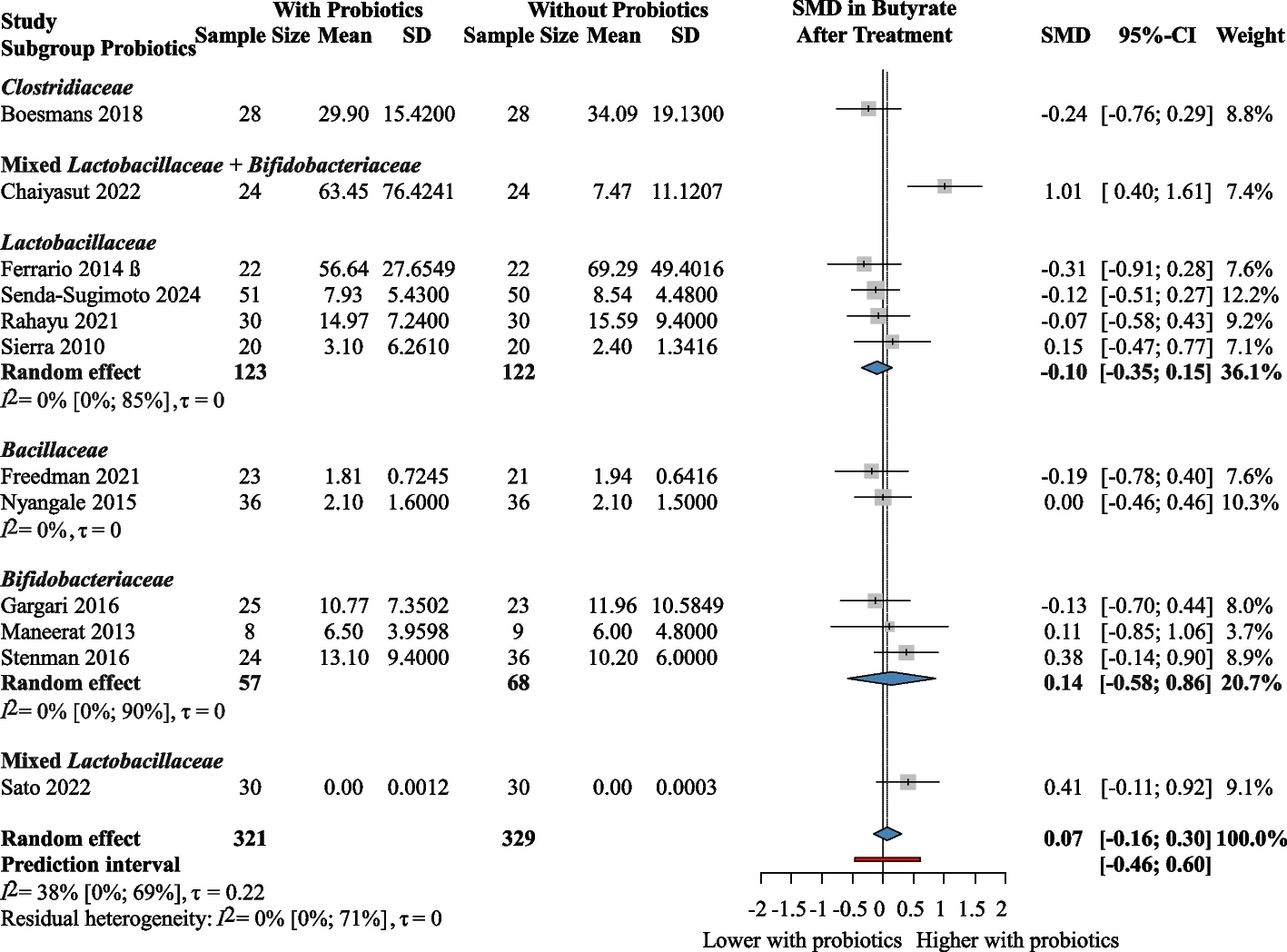
Forest plot of butyrate concentration results of the probiotic composition subgroup analysis. The faecal butyrate concentration was not significantly higher in healthy people receiving probiotic supplementation than in those in the control group, as measured immediately after the treatment period. Studies were subgrouped according to the composition of the probiotic formulation used in each trial. Exact probiotic species/strains are listed in Table 1. CI: confidence interval; SD: standard deviation; SMD: standardized mean difference

We performed subgroup analysis based on the composition of the probiotic formulations used in each study. No probiotic-composition subgroup showed a statistically significant pooled effect on faecal butyrate concentration (Fig. 3). Notably, the mixed *Lactobacillus/Bifidobacterium* formulation subgroup included the trial by Chaiyasut et al. [53], which showed the most pronounced positive study-level effect in favour of probiotics.

As a sensitivity analysis, we performed a separate calculation for data before the treatment, where we did not find any significant difference between the groups either (Supplementary Material 2: Fig. S1) (SMD = 0.02 [(-)0.20 *–* 0.16]). Furthermore, we analysed our data as subgroups based on study design (parallel or crossover design with no data for only the first intervention period) and on the results of risk of bias assessment (high, some concerns, or low risk of bias). These results did not reveal any statistically significant differences between the intervention and control groups. The results of the subgroup analysis based on study design are as follows: parallel design: SMD = 0.19 [(-)0.14 *–* 0.51]; crossover design: SMD = (-)0.16 [(-)0.48 *–* 0.16]. At the same time, subgroups based on risk of bias assessment presented the following results: high risk of bias: SMD = 0.38 [(-)0.14 *–* 0.90]; some concerns: SMD = (-)0.05 [(-)0.31 *–* 0.21]; low risk of bias: SMD = 0.21 [(-)0.64 *–* 1.05] (Supplementary Material 2: Figs. S2-S3).

### The impact of probiotic supplementation on the faecal acetate levels in healthy individuals

**Fig. 4:**
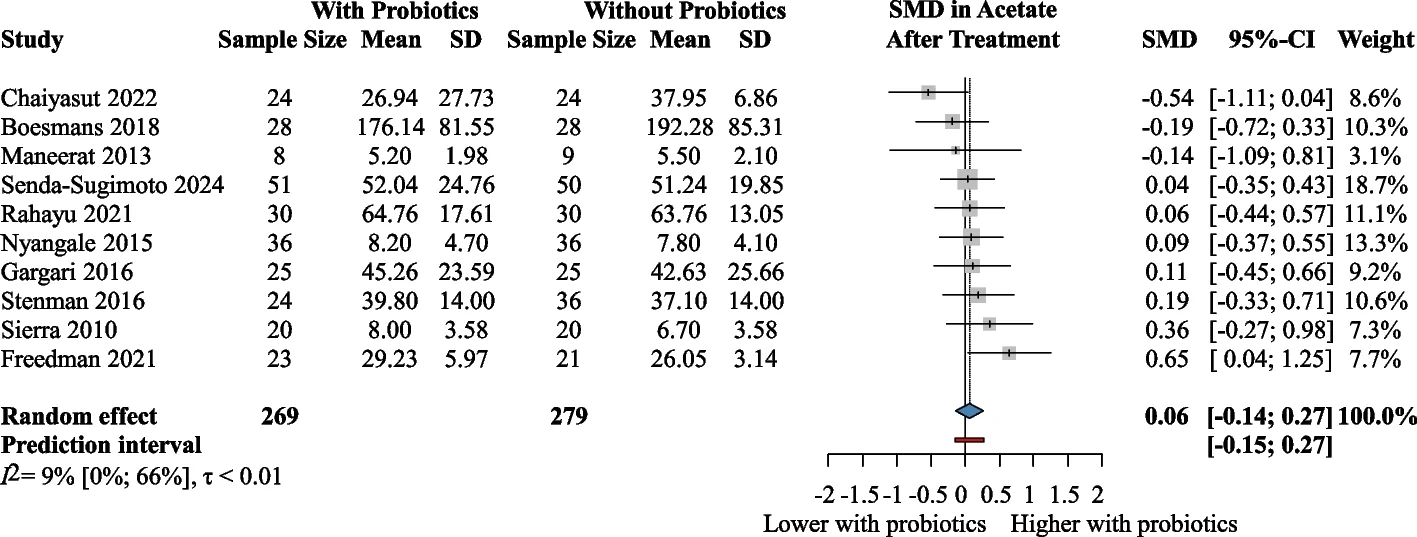
Forest plot of acetate concentration results. The faecal acetate concentration is not significantly higher in healthy people receiving probiotic supplementation than in those in the control group, as measured immediately after the treatment period. CI: confidence interval; SD: standard deviation; SMD: standardized mean difference

**Fig. 5:**
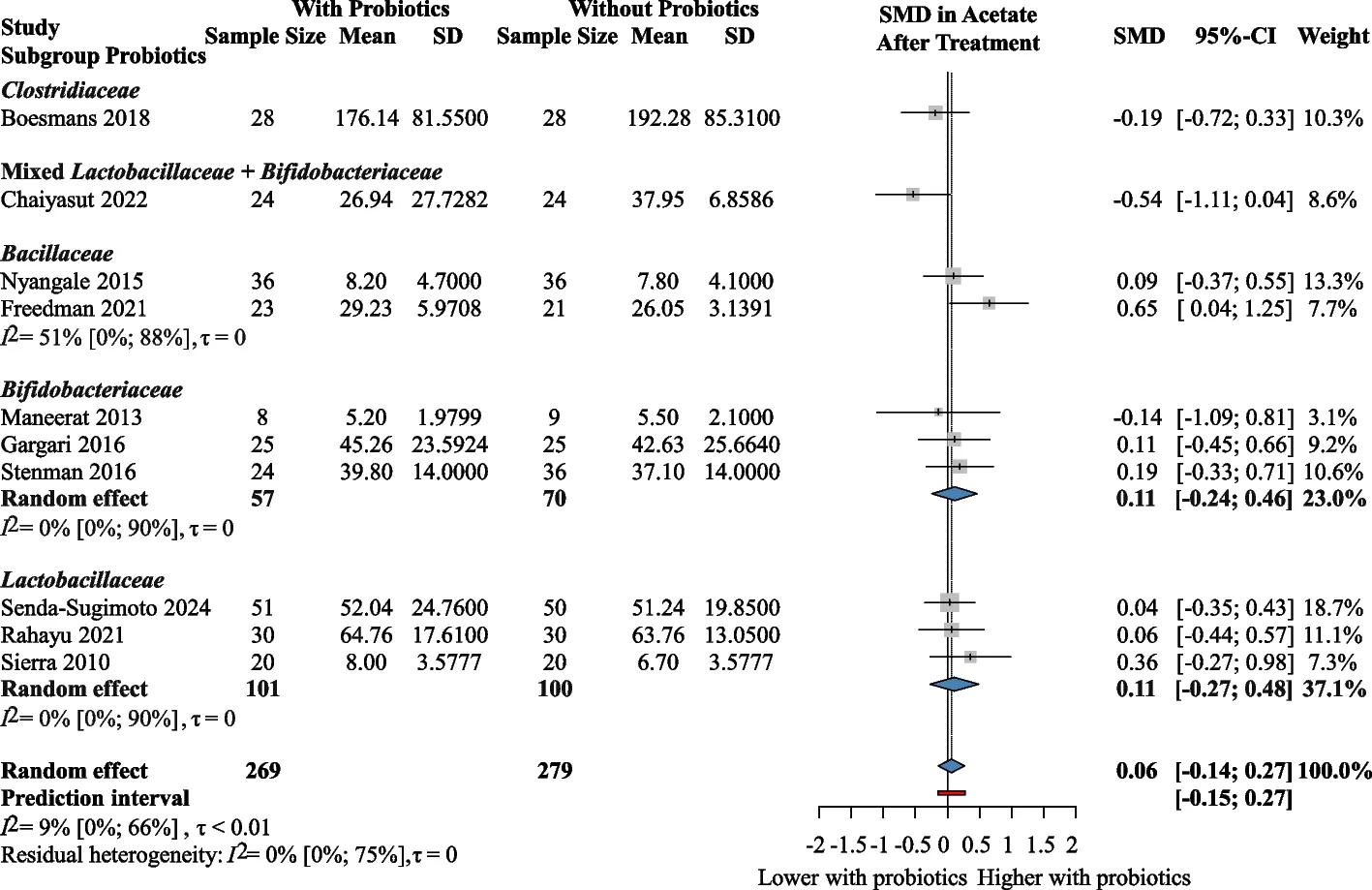
Forest plot of acetate concentration results of probiotic composition subgroup analysis. The faecal acetate concentration is not significantly higher in healthy people receiving probiotic supplementation than in those in the control group as measured immediately after the treatment period. Studies were subgrouped according to the composition of the probiotic formulation used in each trial. Exact probiotic species/strains are listed in Table 1. CI: confidence interval; SD: standard deviation; SMD: standardized mean difference

We identified ten eligible articles with 459 patients in total for the meta-analysis of faecal acetate levels [45, 53, 55, 56, 59–61, 63, 64, 66]. The results are presented in Fig. 4. According to our results, the standardized mean difference in faecal acetate concentrations between the intervention and control groups was 0.06 [(-)0.14–0.27], indicating that the two groups did not significantly and relevantly differ from each other. We did not identify significant baseline or subgroup differences either: baseline: SMD = (-)0.04 [(-)0.21–0.13] (Supplementary Material 2: Fig. S4). Subgroup analysis based on probiotic formulation composition did not reveal any statistically significant differences in faecal acetate concentration (Fig. 5**)**. Investigating the subgroups based on the study design brought the following results: parallel design: SMD = 0.09 [(-)0.23–0.41], crossover design: SMD = 0.01 [(-)0.40–0.41]. Meanwhile, subgroup analysis based on the risk of bias assessment led to similar findings: high risk of bias: SMD = 0.19 [(-)0.33–0.71], some concerns: SMD = (-)0.00 [(-)0.26–0.27], low risk of bias: SMD = 0.11 [(-)0.68–0.90] (Supplementary Material 2: Figs. S5-S6).

### The impact of probiotic supplementation on faecal propionate levels in healthy individuals

**Fig. 6:**
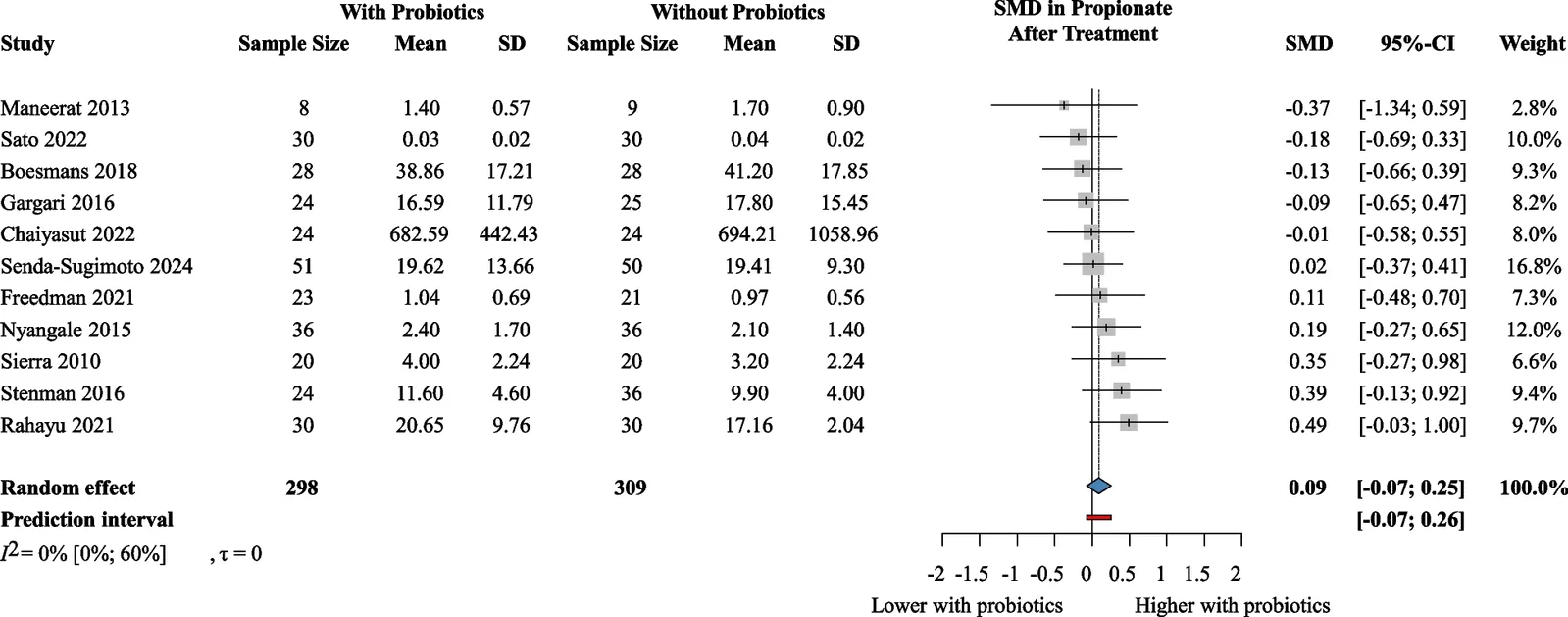
Forest plot of propionate concentration results. The faecal propionate concentration was not significantly higher in healthy people receiving probiotic supplementation than in those in the control group, as measured immediately after the treatment period. CI: confidence interval; SD: standard deviation; SMD: standardized mean difference

**Fig. 7:**
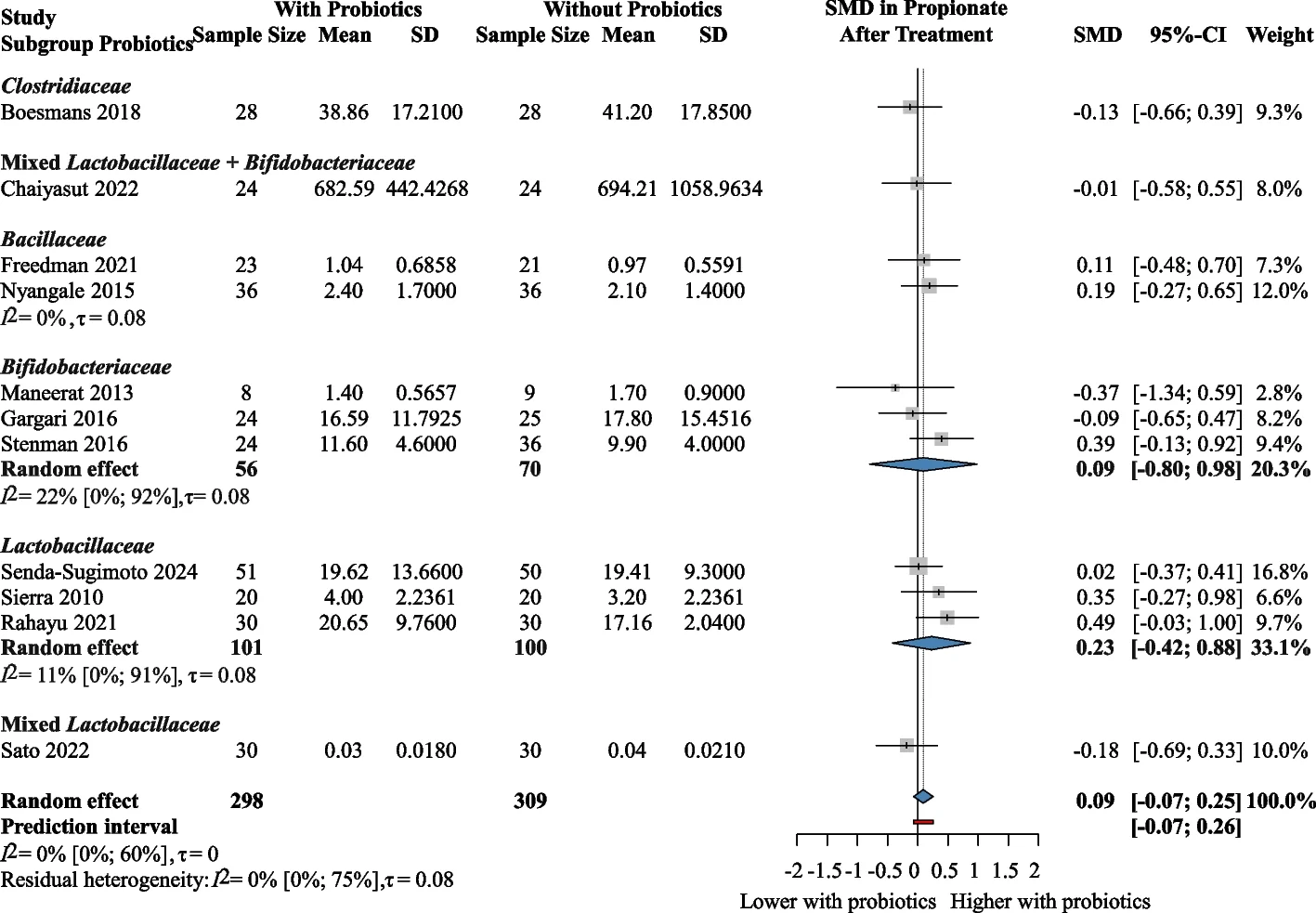
Forest plot of propionate concentration results of the probiotic composition subgroup analysis. The faecal propionate concentration was not significantly higher in healthy people receiving probiotic supplementation than in those in the control group as measured immediately after the treatment period. Studies were subgrouped according to the composition of the probiotic formulation used in each trial. Exact probiotic species/strains are listed in Table 1. CI: confidence interval; SD: standard deviation; SMD: standardized mean difference

Eleven eligible articles with 519 participants provided data on faecal propionate levels for quantitative analysis [45, 53, 55, 56, 59–61, 63, 64, 66, 68]. The results are presented in Fig. 6. According to our findings, probiotic supplementation did not result in a significantly different faecal propionate concentration than that in the control group. The standardized mean difference in propionate levels between the intervention and control groups was 0.09 [(-)0.07–0.25]. Subgroup analysis based on probiotic formulation composition likewise did not reveal any statistically significant effect on faecal propionate concentration (Fig. 7).

The analysis of the baseline values revealed no significant or relevant differences between the groups (Supplementary Material 2: Fig. S7) (SMD = (-)0.01 [(-)0.18–0.15]). Additional subgroup analyses based on study design and risk of bias assessment can be found in Supplementary Material 2: Figs. S8-S9, with no significant or relevant differences. parallel design: SMD = 0.13 [(-)0.09–0.351], crossover design: SMD = 0.01 [(-)0.44–0.46]; high risk of bias: SMD = 0.39 [(-)0.13–0.92], some concerns: SMD = (-)0.04 [(-)0.25–0.17], low risk of bias: SMD = 0.24 [(-)0.13–0.62].

### Publication bias and leave-one-out analysis

Publication bias and leave-out analyses did not raise serious concerns or influential articles regarding the results of our meta-analyses (Supplementary Material 2: Figs. S10-S15).

### The impact of probiotic supplementation on faecal short-chain fatty acid concentrations in generally healthy populations

Supplementary Material 1: Table S3 summarizes the results of all SCFA levels from the studies, including those not included into the meta-analysis. Only two articles reported a significant difference between the probiotic and control groups as measured after the intervention. Chaiyasut et al. [53] reported that a probiotic mixture of *Lactobacillus paracasei* HII01, *Bifidobacterium breve,* and *Bifidobacterium longum* resulted in a significantly higher faecal butyrate concentration compared with placebo. They also reported that faecal propionate levels increased statistically significantly in the intervention group after treatment; however, this difference in faecal propionate concentration was not statistically significant compared with that in the control group. In the study of Freedman et al. [56] faecal acetate levels were found to be significantly higher in the probiotic group. However, the authors highlighted that this was caused mainly by the observed decrease in faecal acetate levels in the placebo group and not by the supplementation with *Bacillus subtilis strain* DE111. Other studies reported no significant difference in faecal SCFA concentrations between the intervention and control groups [45, 54, 55, 58–66, 68–70]. Ferrario et al. [58] reported a statistically significant decrease in faecal butyrate levels after intervention with *Lactobacillus paracasei DG.*

### Risk of bias assessment

The results of the risk of bias assessment are detailed in Supplementary Material 1: Tables S4-S6 and Supplementary Material 2: Figs. S16-S18. The overall risk of bias was low to high for the studies included in the systematic review and meta-analysis. The high risk of bias was caused by the high dropout rate [64], very low number of participants [54], and sub-grouped data reporting [70]. Some concerns arose mainly in crossover studies, where separate data after each period were not available, and/or there were a high dropout rate [45, 58–62]. Other parallel studies reported confusing data where baseline values were not accurately reported [65] or reported a moderate dropout rate [57].

On the basis of the GRADE assessment, the quality of evidence for the meta-analyses was low (Supplementary Material 1: Table S7).

## Discussion

This study represents, to our knowledge, the first meta-analysis consolidating the outcomes of existing RCTs investigating the effect of probiotic supplementation on faecal SCFA levels in generally healthy populations. The outcomes of our quantitative data synthesis do not demonstrate a statistically significant effect of probiotics on faecal SCFA concentrations in healthy individuals compared with control. Therefore, the currently available evidence from randomized controlled trials does not support a measurable increase in faecal SCFA concentrations in healthy individuals following probiotic supplementation. This interpretation should nevertheless remain cautious, as the certainty of evidence was low according to the GRADE assessment (Supplementary Material 1: Table S7).

These findings are also in line with several of our previous evidence syntheses on probiotics, although those addressed different outcomes and populations. In a recent systematic review and meta-analysis, we revealed that probiotics do not significantly alter gut microbiome in healthy individuals [74] and do not effectively maintain gut microbiome diversity, during antibiotic therapy [75]. In addition, probiotics have been found to be ineffective in preventing *Clostridioides difficile* infection [76] and in altering zonulin levels in healthy individuals [8].

This question remains clinically relevant because probiotic products are widely promoted to healthy individuals with claims related to gut health and microbial function [3]. One proposed mechanism underlying such benefits is the modulation of faecal SCFA-related outcomes [77]. Therefore, our aim was to assess the effects of probiotic supplements on faecal SCFA levels in healthy humans. The findings from our systematic review and meta-analysis do not support the use of probiotic supplementation to increase faecal SCFA levels in healthy individuals. The meta-analysis of faecal butyrate, acetate, and propionate concentrations did not show a statistically significant effect of probiotics on faecal SCFA levels in generally healthy human populations. Because faecal SCFA concentrations are influenced by microbial production, host absorption, intestinal transit, and luminal handling, they cannot be used to conclude directly that intestinal SCFA production itself was unchanged.

There could be several factors contributing to the lack of detectable effect. First, probiotic effects are highly strain-specific and influenced by dose and duration of probiotic supplementation. Second, we could not detect a clearly effective combination of these factors owing to the low number of studies with similar protocols for performing a specified subgroup analysis. Third, not all commonly used probiotic strains are direct SCFA producers. Promising species could be well-known butyrate producers, such as *Faecalibacterium, Roseburia, Eubacterium, Anaerostipes, Coprococcus, Subdoligranulum, and Anaerobutyricum* [9, 78]. Although *Butyricicoccus pullicaecorum*, identified as a butyrate-producer, exhibited robust tolerance and was still metabolically active after upper gastrointestinal conditions producing SCFAs in vitro [79], the study of Boesmans et al. did not reveal any significant or relevant effects of these bacteria on faecal SCFA concentrations in humans [59].

*Bacillus* species do not produce butyrate directly, but they are believed to exert indirect and modulating effects on the gut microbiota, stimulating the production of SCFAs in other microbial taxa [80, 81]. However, this effect on faecal SCFA levels was not observed in the randomized controlled human trials that used *Bacillus* strains as an intervention in our review, even in the case where the level of *Faecalibacterium prausnitzii* was otherwise increased [56, 60]. Most of the studies we included investigated *Lactobacillus* and/or *Bifidobacterium* species, which have been shown to be able to produce SCFAs under several conditions [77, 82]. However, this was not reflected in a statistically significant increase in faecal SCFA concentrations compared with placebo in the randomized controlled trials included in our review.

Individual characteristics may also modify the response to supplementation. These include age, a gut microbiota composition with higher or lower SCFA production capacity and a bacterial cross-feeding mechanism, all of which may influence the results, on which probiotics may have only a limited effect [83].

Alba et al. reported beneficial effects of probiotic supplementation in formula-fed infants, which were able to significantly increase faecal SCFA concentrations compared with those in the control group [72]. This finding suggests that interventions that target immature microbiota could potentially be more effective. Notably, however, this was not observed in the studies of Hiraku et al. and Bazanella et al. who investigated newborns as well [70, 71].

Of note, Chaiyasut et al. [53] reported the most pronounced increase in faecal butyrate among the included studies. This low-risk-of-bias trial used a mixed probiotic formulation containing *Lactobacillus paracasei* HII01, *Bifidobacterium breve*, and *Bifidobacterium longum*. Although this finding derives from a single study and should be interpreted cautiously, it may support the possibility that multi-strain formulations allowing cross-feeding interactions warrant further investigation. Freedman et al. [56] found higher acetate levels in the probiotic group, although the authors noted that this appeared to be driven mainly by a decrease in the placebo group. By contrast, Ferrario et al. [58] reported a decrease in butyrate after probiotic intervention. Taken together, these findings suggest heterogeneity of response rather than a uniform effect across healthy populations.

In the study of Lee et al. subjects receiving both viable probiotics had significantly higher acetate and butyrate concentrations in faeces than before probiotic administration, although the between-group comparison showed no difference from the control group [84]. Nakamura et al. reported their results by grouping participants into responders and non-responders rather than presenting the outcome in the original randomized probiotic-versus-control group format. According, although responders showed greater increases in propionate and butyrate abundance after *B. longum BB536* intake, a direct comparison with the control group was not possible [67].

Baseline faecal SCFA concentration may also partly explain the absence of a uniform overall observed effect. Gargari et al. and Ferrario et al. suggested that probiotic supplementation did not simply increase faecal SCFA concentrations across all participants; rather, participants with higher baseline concentrations tended to show decreases after intervention, whereas those with lower baseline concentrations tended to show increases [45, 58]. This pattern may suggest a normalization-like effect, although this interpretation should be considered cautiously [45, 58].

It is also important to interpret these findings in the broader context of SCFA biology. Although SCFAs are widely regarded as beneficial microbial metabolites, faecal SCFA levels do not always show a straightforward relationship with health status. Epidemiologic research and murine models have repeatedly demonstrated that increased faecal SCFA concentrations are positively correlated with body weight and increase in response to diets high in calories. Moreover, leanness has been linked to reduced faecal SCFA levels [85–88]. However, clear cut-off points are not yet defined.

It is important to highlight that diet also plays a crucial role in determining the types and amounts of SCFAs produced. The consumption of several dietary fibres can especially contribute to variations in SCFA concentrations, making them promising intervention points alone or in combination with a symbiotic [83, 89, 90]. However, the studies included in our review that investigated prebiotics and/or synbiotics did not report a statistically significant effect of these products on faecal butyrate, acetate, or propionate concentrations [61, 62, 64].

### Implications for practice and research

According to our findings, probiotics did not show statistically significant effect on faecal SCFA levels in generally healthy individuals and therefore cannot currently be recommended with the specific aim of increasing faecal SCFA levels.

Additional assessment of the associations between possible clinical manifestations and SCFA levels as predictive and/or preventive factors and how different interventions affect them will result in more comprehensive knowledge about the role of microbial metabolites in health and disease. The standardization of faecal SCFA concentration measurement methods with optimal values along with proper microbiome composition analysis are key elements for achieving greater data uniformity with a wider understanding of the role of gut microbe's function and possible cross-talks between species. Determining individual baseline faecal SCFA values can help understand who might benefit from probiotic supplementation, although additional good-quality studies are needed to understand the underlying mechanisms in greater depth. Further research on additional strains and their combinations is also needed to better understand the effects of different bacteria on SCFA levels.

### Strengths and limitations

Our study represents the highest level of presently available evidence by exclusively incorporating RCTs in our comprehensive review and meta-analysis. Additionally, to the best of our knowledge, this is the first meta-analysis and the most extensive review on this topic to date. However, caution is warranted in interpreting our results, given the relatively small sample sizes and the restricted number of eligible studies. Some data could not undergo quantitative synthesis because of insufficient reporting. The included studies varied greatly regarding the strains, dose, and duration of probiotic supplementation, which prevented us from performing targeted analyses of the effects of special species sub-groups. Importantly, the present review focused on faecal SCFA concentrations, which represent only the fraction of SCFAs remaining after colonic absorption and therefore do not directly quantify total intestinal SCFA production. Faecal SCFA levels should thus be interpreted as a practical but indirect proxy of microbial fermentation.

## Conclusions

The consolidated results from the existing RCTs do not provide sufficient support for recommending probiotic supplementation to increase faecal SCFA concentrations in generally healthy populations. The meta-analyses of butyrate, acetate, and propionate levels revealed no significant effect of probiotics compared with placebo. Although we were unable to conduct a quantitative analysis of all the identified results, the articles included in the systematic review confirmed and strengthened our findings. Further research on additional strains and combinations, along with microbiome and initial SCFA analysis, is needed to better understand the effects of different bacteria on SCFA production and faecal SCFA concentrations and to identify individuals who might benefit from probiotic supplementation.

## Supplementary Information

Supplementary Material 1: Tables. Table S1 PRISMA checklist 2020. Table S2 Search key. Table S3 Changes in the faecal SCFA levels as measured after the intervention period.. Table S4 Risk of bias assessment for parallel design studies—Assignment to intervention (the 'intention-to-treat' effect). Table S5 Risk of bias assessment for parallel design studies—Adhering to intervention (the 'per-protocol' effect). Table S6 Risk of bias assessment for crossover design studies—Adhering to intervention (the 'per-protocol' effect). Table S7 GRADE assessment for the meta-analyses of faecal butyrate, acetate, and propionate concentrations.

Supplementary Material 2: Figures. Fig. S1 Additional sensitivity analysis for the baseline values of faecal butyrate concentration. Fig. S2 Additional sensitivity analysis for the subgroups based on study design for faecal butyrate concentration. Fig. S3 Additional sensitivity analysis for the subgroups based on risk of bias assessment for faecal butyrate concentration. Fig. S4 Additional sensitivity analysis for the baseline values of faecal acetate concentration. Fig. S5 Additional sensitivity analysis for the subgroups based on study design for faecal acetate concentration. Fig. S6 Additional sensitivity analysis for the subgroups based on risk of bias assessment for faecal acetate concentration. Fig. S7 Additional sensitivity analysis for the baseline values of faecal propionate concentration. Fig. S8 Additional sensitivity analysis for the subgroups based on study design for faecal propionate concentration. Fig. S9 Additional sensitivity analysis for the subgroups based on risk of bias assessment for faecal propionate concentration. Fig. S10 Funnel plot representing publication bias for faecal butyrate concentration. Fig. S11 Additional leave-one-out analysis for faecal butyrate concentration. Fig. S12 Funnel plot representing publication bias for faecal acetate concentration. Fig. S13 Additional leave-one-out analysis for faecal acetate concentration. Fig. S14 Funnel plot representing publication bias for faecal propionate concentration. Fig. S15 Additional leave-one-out analysis for faecal propionate concentration. Fig. S16 Risk of bias assessment for parallel design studies—Assignment to intervention (the 'intention-to-treat' effect). Fig. S17 Risk of bias assessment for parallel design studies—Adhering to intervention (the 'per-protocol' effect). Fig. S18 Risk of bias assessment for crossover design studies—Adhering to intervention (the 'per-protocol' effect).

## Data Availability

All datasets used in this study can be found in the full-text articles included in the systematic review and meta-analysis.

## Abbreviations

CFU: Colony Forming Unit
CI: Confidence Interval
CRC: Colorectal Cancer
GRADE: The Grading of Recommendations, Assessment, Development and Evaluation
SMD: Standardized Mean Difference
PRISMA: Preferred Reporting Items for Systematic Reviews and Meta-Analyses
PROSPERO: International Prospective Register of Systematic Reviews
RCT: Randomized Controlled Trial
RoB: Risk of Bias
SCFA: Short-Chain Fatty Acid
SD: Standard Deviation
UK: United Kingdom
USA: United States of America
