# Supplementary material for: Effects of probiotic supplementation on faecal short-chain fatty acid concentrations in healthy individuals: a systematic review and meta-analysis of randomized controlled trials
Source: Nutr J. 2026 Jul 13;25:106. doi: 10.1186/s12937-026-01364-0 (PMC13644228; doi:10.1186/s12937-026-01364-0)
Supplement: Supplementary file 1 [file 12937_2026_1364_MOESM1_ESM.docx]

**Supplementary Material 1 – Tables**

**Contents**

[**Table S1.** PRISMA checklist 2020	2](#_Toc203166150)

[**Table S2.** Search key	6](#_Toc203166151)

[**Table S3.** Changes in the faecal SCFA levels as measured after the intervention period.	10](#_Toc203166152)

[**Table S4.** Risk of bias assessment for parallel design studies - Assignment to intervention (the 'intention-to-treat' effect).	12](#_Toc203166153)

[**Table S5.** Risk of bias assessment for parallel design studies - Adhering to intervention (the 'per-protocol' effect).	13](#_Toc203166154)

[**Table S6.** Risk of bias assessment for crossover design studies - Adhering to intervention (the 'per-protocol' effect).	14](#_Toc203166155)

[**Table S7.** GRADE assessment for the meta-analyses of faecal butyrate, acetate, and propionate concentrations.	15](#_Toc203166156)

# **Table S1.** PRISMA checklist 2020

| **Section and Topic** | **Item #** | **Checklist item** | **Location where item is reported** |
| --- | --- | --- | --- |
| **TITLE** | | |  |
| Title | 1 | Identify the report as a systematic review. | Title page |
| **ABSTRACT** | | |  |
| Abstract | 2 | See the PRISMA 2020 for Abstracts checklist. | Page 3 |
| **INTRODUCTION** | | |  |
| Rationale | 3 | Describe the rationale for the review in the context of existing knowledge. | Page 4-6 |
| Objectives | 4 | Provide an explicit statement of the objective(s) or question(s) the review addresses. | Page 6 |
| **METHODS** | | |  |
| Eligibility criteria | 5 | Specify the inclusion and exclusion criteria for the review and how studies were grouped for the syntheses. | Page 6-7 |
| Information sources | 6 | Specify all databases, registers, websites, organisations, reference lists and other sources searched or consulted to identify studies. Specify the date when each source was last searched or consulted. | Page 7 |
| Search strategy | 7 | Present the full search strategies for all databases, registers, and websites, including any filters and limits used. | Page 7 and S2 Table |
| Selection process | 8 | Specify the methods used to decide whether a study met the inclusion criteria of the review, including how many reviewers screened each record and each report retrieved, whether they worked independently, and if applicable, details of automation tools used in the process. | Page 6-8 and Page 13 (Figure 1) |
| Data collection process | 9 | Specify the methods used to collect data from reports, including how many reviewers collected data from each report, whether they worked independently, any processes for obtaining or confirming data from study investigators, and if applicable, details of automation tools used in the process. | Page 8 |
| Data items | 10a | List and define all outcomes for which data were sought. Specify whether all results that were compatible with each outcome domain in each study were sought (e.g., for all measures, time points, analyses), and if not, the methods used to decide which results to collect. | Page 8 |
|  | 10b | List and define all other variables for which data were sought (e.g., participant and intervention characteristics, funding sources). Describe any assumptions made about any missing or unclear information. | Page 8 and Page 14-17 (Table 1) |
| Study risk of bias assessment | 11 | Specify the methods used to assess risk of bias in the included studies, including details of the tool(s) used, how many reviewers assessed each study and whether they worked independently, and if applicable, details of automation tools used in the process. | Page 10 |
| Effect measures | 12 | Specify for each outcome the effect measure(s) (e.g., risk ratio, mean difference) used in the synthesis or presentation of results. | Page 8-10 |
| Synthesis methods | 13a | Describe the processes used to decide which studies were eligible for each synthesis (e.g., tabulating the study intervention characteristics and comparing against the planned groups for each synthesis (item #5)). | Page 8-10 |
|  | 13b | Describe any methods required to prepare the data for presentation or synthesis, such as handling of missing summary statistics, or data conversions. | Page 8-10 |
|  | 13c | Describe any methods used to tabulate or visually display results of individual studies and syntheses. | Page 8-10 |
|  | 13d | Describe any methods used to synthesize results and provide a rationale for the choice(s). If meta-analysis was performed, describe the model(s), method(s) to identify the presence and extent of statistical heterogeneity, and software package(s) used. | Page 8-10 |
|  | 13e | Describe any methods used to explore possible causes of heterogeneity among study results (e.g., subgroup analysis, meta-regression). | Page 8-10 |
|  | 13f | Describe any sensitivity analyses conducted to assess robustness of the synthesized results. | Page 8-10 |
| Reporting bias assessment | 14 | Describe any methods used to assess risk of bias due to missing results in a synthesis (arising from reporting biases). | Page 10 |
| Certainty assessment | 15 | Describe any methods used to assess certainty (or confidence) in the body of evidence for an outcome. | Page 10-11 |
| **RESULTS** | | |  |
| Study selection | 16a | Describe the results of the search and selection process, from the number of records identified in the search to the number of studies included in the review, ideally using a flow diagram. | Page 6-8 and Page 10-13 (Figure 1) |
|  | 16b | Cite studies that might appear to meet the inclusion criteria, but which were excluded, and explain why they were excluded. | Page 6-8 and Page 10-13 (Figure 1) |
| Study characteristics | 17 | Cite each included study and present its characteristics. | Page 11-17 (Table 1) |
| Risk of bias in studies | 18 | Present assessments of risk of bias for each included study. | Page 24-25 and S4-6 Tables and S16-18 Figure |
| Results of individual studies | 19 | For all outcomes, present, for each study: (a) summary statistics for each group (where appropriate) and (b) an effect estimate and its precision (e.g. confidence/credible interval), ideally using structured tables or plots. | Page 18-24 Figure 2-7 and S1-9 Figure and S3 Table |
| Results of syntheses | 20a | For each synthesis, briefly summarise the characteristics and risk of bias among contributing studies. | Page 24-25 and S4-6 Tables and S16-18 Figure |
|  | 20b | Present results of all statistical syntheses conducted. If meta-analysis was done, present for each the summary estimate and its precision (e.g., confidence/credible interval) and measures of statistical heterogeneity. If comparing groups, describe the direction of the effect. | Page 18-24 Figure 2-7 and S1-9 Figure |
|  | 20c | Present results of all investigations of possible causes of heterogeneity among study results. | Page 24 and S10-15 Figure |
|  | 20d | Present results of all sensitivity analyses conducted to assess the robustness of the synthesized results. | Page 18-24 and S1-9 Figure |
| Reporting biases | 21 | Present assessments of risk of bias due to missing results (arising from reporting biases) for each synthesis assessed. | Page 24-25 and S4-6 Tables and S16-18 Figure |
| Certainty of evidence | 22 | Present assessments of certainty (or confidence) in the body of evidence for each outcome assessed. | Page 25 and S7 Table |
| **DISCUSSION** | | |  |
| Discussion | 23a | Provide a general interpretation of the results in the context of other evidence. | Page 25-27 |
|  | 23b | Discuss any limitations of the evidence included in the review. | Page 28 |
|  | 23c | Discuss any limitations of the review processes used. | Page 28 |
|  | 23d | Discuss implications of the results for practice, policy, and future research. | Page 28 |
| **OTHER INFORMATION** | | |  |
| Registration and protocol | 24a | Provide registration information for the review, including register name and registration number, or state that the review was not registered. | Page 7-8 |
|  | 24b | Indicate where the review protocol can be accessed, or state that a protocol was not prepared. | Page 7-8 |
|  | 24c | Describe and explain any amendments to information provided at registration or in the protocol. | Page 7-8 |
| Support | 25 | Describe sources of financial or non-financial support for the review, and the role of the funders or sponsors in the review. | Title Page (Page 1) |
| Competing interests | 26 | Declare any competing interests of review authors. | Page 30 |
| Availability of data, code and other materials | 27 | Report which of the following are publicly available and where they can be found: template data collection forms; data extracted from included studies; data used for all analyses; analytic code; any other materials used in the review. | Page 29-30 |

# **Table S2.** Search key

| **Search key** | (normal OR general OR healthy) **AND** (population OR participant OR participants OR volunteer OR volunteers OR subject OR subjects OR adult OR adults OR adolescent OR adolescents OR child OR children OR infant OR infants OR newborn OR newborns OR birth cohort OR pediatric* OR elderly OR elders) **AND** (probiotic OR probiotic* OR bifidobac* OR lactobac* OR escherichia OR streptococcus OR saccharomyces OR bacillus OR pediococc* OR leuconostoc) **AND** random* |
| --- | --- |
| **MEDLINE (Expanded)** | ("normal"[All Fields] OR "normalisation"[All Fields] OR "normalisations"[All Fields] OR "normalise"[All Fields] OR "normalised"[All Fields] OR "normalises"[All Fields] OR "normalising"[All Fields] OR "normalization"[All Fields] OR "normalizations"[All Fields] OR "normalize"[All Fields] OR "normalized"[All Fields] OR "normalizer"[All Fields] OR "normalizers"[All Fields] OR "normalizes"[All Fields] OR "normalizing"[All Fields] OR "normally"[All Fields] OR "normals"[All Fields] OR ("drugs, generic"[MeSH Terms] OR ("drugs"[All Fields] AND "generic"[All Fields]) OR "generic drugs"[All Fields] OR "generic"[All Fields] OR "family characteristics"[MeSH Terms] OR ("family"[All Fields] AND "characteristics"[All Fields]) OR "family characteristics"[All Fields] OR "generation"[All Fields] OR "generations"[All Fields] OR "general"[All Fields] OR "general s"[All Fields] OR "generalisability"[All Fields] OR "generalisable"[All Fields] OR "generalisation"[All Fields] OR "generalization, psychological"[MeSH Terms] OR ("generalization"[All Fields] AND "psychological"[All Fields]) OR "psychological generalization"[All Fields] OR "generalization"[All Fields] OR "generalisations"[All Fields] OR "generalise"[All Fields] OR "generalised"[All Fields] OR "generalises"[All Fields] OR "generalisibility"[All Fields] OR "generalising"[All Fields] OR "generalities"[All Fields] OR "generality"[All Fields] OR "generalizability"[All Fields] OR "generalizable"[All Fields] OR "generalizations"[All Fields] OR "generalize"[All Fields] OR "generalized"[All Fields] OR "generalizes"[All Fields] OR "generalizing"[All Fields] OR "generally"[All Fields] OR "generals"[All Fields] OR "generate"[All Fields] OR "generated"[All Fields] OR "generates"[All Fields] OR "generating"[All Fields] OR "generation s"[All Fields] OR "generational"[All Fields] OR "generative"[All Fields] OR "generatively"[All Fields] OR "generativity"[All Fields] OR "generator"[All Fields] OR "generator s"[All Fields] OR "generators"[All Fields] OR "generically"[All Fields] OR "genericity"[All Fields] OR "generics"[All Fields]) OR ("healthies"[All Fields] OR "healthy"[All Fields])) AND ("populate"[All Fields] OR "populated"[All Fields] OR "populates"[All Fields] OR "populating"[All Fields] OR "population"[MeSH Terms] OR "population"[All Fields] OR "population groups"[MeSH Terms] OR ("population"[All Fields] AND "groups"[All Fields]) OR "population groups"[All Fields] OR "populations"[All Fields] OR "population s"[All Fields] OR "populational"[All Fields] OR "populous"[All Fields] OR ("participant"[All Fields] OR "participant s"[All Fields] OR "participants"[All Fields] OR "participate"[All Fields] OR "participated"[All Fields] OR "participates"[All Fields] OR "participating"[All Fields] OR "participation"[All Fields] OR "participations"[All Fields] OR "participative"[All Fields] OR "participator"[All Fields] OR "participators"[All Fields]) OR ("participant"[All Fields] OR "participant s"[All Fields] OR "participants"[All Fields] OR "participate"[All Fields] OR "participated"[All Fields] OR "participates"[All Fields] OR "participating"[All Fields] OR "participation"[All Fields] OR "participations"[All Fields] OR "participative"[All Fields] OR "participator"[All Fields] OR "participators"[All Fields]) OR ("volunteer s"[All Fields] OR "volunteered"[All Fields] OR "volunteers"[MeSH Terms] OR "volunteers"[All Fields] OR "volunteer"[All Fields] OR "volunteering"[All Fields]) OR ("volunteer s"[All Fields] OR "volunteered"[All Fields] OR "volunteers"[MeSH Terms] OR "volunteers"[All Fields] OR "volunteer"[All Fields] OR "volunteering"[All Fields]) OR ("subject"[All Fields] OR "subject s"[All Fields] OR "subjects"[All Fields] OR "subjects s"[All Fields]) OR ("subject"[All Fields] OR "subject s"[All Fields] OR "subjects"[All Fields] OR "subjects s"[All Fields]) OR ("adult"[MeSH Terms] OR "adult"[All Fields] OR "adults"[All Fields] OR "adult s"[All Fields]) OR ("adult"[MeSH Terms] OR "adult"[All Fields] OR "adults"[All Fields] OR "adult s"[All Fields]) OR ("adolescences"[All Fields] OR "adolescency"[All Fields] OR "adolescent"[MeSH Terms] OR "adolescent"[All Fields] OR "adolescence"[All Fields] OR "adolescents"[All Fields] OR "adolescent s"[All Fields]) OR ("adolescences"[All Fields] OR "adolescency"[All Fields] OR "adolescent"[MeSH Terms] OR "adolescent"[All Fields] OR "adolescence"[All Fields] OR "adolescents"[All Fields] OR "adolescent s"[All Fields]) OR ("child"[MeSH Terms] OR "child"[All Fields] OR "children"[All Fields] OR "child s"[All Fields] OR "children s"[All Fields] OR "childrens"[All Fields] OR "childs"[All Fields]) OR ("child"[MeSH Terms] OR "child"[All Fields] OR "children"[All Fields] OR "child s"[All Fields] OR "children s"[All Fields] OR "childrens"[All Fields] OR "childs"[All Fields]) OR ("infant"[MeSH Terms] OR "infant"[All Fields] OR "infants"[All Fields] OR "infant s"[All Fields]) OR ("infant"[MeSH Terms] OR "infant"[All Fields] OR "infants"[All Fields] OR "infant s"[All Fields]) OR ("infant, newborn"[MeSH Terms] OR ("infant"[All Fields] AND "newborn"[All Fields]) OR "newborn infant"[All Fields] OR "newborn"[All Fields] OR "newborns"[All Fields] OR "newborn s"[All Fields]) OR ("infant, newborn"[MeSH Terms] OR ("infant"[All Fields] AND "newborn"[All Fields]) OR "newborn infant"[All Fields] OR "newborn"[All Fields] OR "newborns"[All Fields] OR "newborn s"[All Fields]) OR ("birth cohort"[MeSH Terms] OR ("birth"[All Fields] AND "cohort"[All Fields]) OR "birth cohort"[All Fields]) OR "pediatric*"[All Fields] OR ("aged"[MeSH Terms] OR "aged"[All Fields] OR "elderly"[All Fields] OR "elderlies"[All Fields] OR "elderly s"[All Fields] OR "elderlys"[All Fields]) OR ("elder s"[All Fields] OR "elders"[All Fields] OR "sambucus"[MeSH Terms] OR "sambucus"[All Fields] OR "elder"[All Fields])) AND ("probiotic s"[All Fields] OR "probiotical"[All Fields] OR "probiotics"[MeSH Terms] OR "probiotics"[All Fields] OR "probiotic"[All Fields] OR "probiotic*"[All Fields] OR "bifidobac*"[All Fields] OR "lactobac*"[All Fields] OR ("escherichia"[MeSH Terms] OR "escherichia"[All Fields] OR "escherichiae"[All Fields]) OR ("streptococcus"[MeSH Terms] OR "streptococcus"[All Fields]) OR ("saccharomyces"[MeSH Terms] OR "saccharomyces"[All Fields] OR "saccharomyce"[All Fields]) OR ("bacillu"[All Fields] OR "bacillus"[MeSH Terms] OR "bacillus"[All Fields]) OR "pediococc*"[All Fields] OR ("leuconostoc"[MeSH Terms] OR "leuconostoc"[All Fields] OR "leuconostocs"[All Fields])) AND "random*"[All Fields] |
| **EMBASE (Expanded)** | (normal OR general OR healthy) AND ('population'/exp OR population OR participant OR participants OR 'volunteer'/exp OR volunteer OR 'volunteers'/exp OR volunteers OR subject OR subjects OR 'adult'/exp OR adult OR 'adults'/exp OR adults OR 'adolescent'/exp OR adolescent OR 'adolescents'/exp OR adolescents OR 'child'/exp OR child OR 'children'/exp OR children OR 'infant'/exp OR infant OR 'infants'/exp OR infants OR 'newborn'/exp OR newborn OR newborns OR 'birth cohort'/exp OR 'birth cohort' OR (('birth'/exp OR birth) AND ('cohort'/exp OR cohort)) OR pediatric* OR 'elderly'/exp OR elderly OR elders) AND ('probiotic'/exp OR probiotic OR probiotic* OR bifidobac* OR lactobac* OR 'escherichia'/exp OR escherichia OR 'streptococcus'/exp OR streptococcus OR 'saccharomyces'/exp OR saccharomyces OR 'bacillus'/exp OR bacillus OR pediococc* OR 'leuconostoc'/exp OR leuconostoc) AND random* |
| **Cochrane Register of Controlled Trials (CENTRAL) (Expanded)** | (normal OR general OR healthy) AND (population OR participant OR participants OR volunteer OR volunteers OR subject OR subjects OR adult OR adults OR adolescent OR adolescents OR child OR children OR infant OR infants OR newborn OR newborns OR birth cohort OR pediatric* OR elderly OR elders) AND (probiotic OR probiotic* OR bifidobac* OR lactobac* OR escherichia OR streptococcus OR saccharomyces OR bacillus OR pediococc* OR leuconostoc) AND random* |

# **Table S3.** Changes in the faecal SCFA levels as measured after the intervention period.

| **Study** | **Butyrate** | **Acetate** | **Propionate** |
| --- | --- | --- | --- |
| Alba (2024) |  |  |  |
| Bazanella (2017) |  | N.A. |  |
| Boesmans (2018) |  |  |  |
| Chaiyasut (2022) |  |  |  |
| Childs (2014) |  |  |  |
| Ferrario (2014) |  |  |  |
| Freedman (2021) |  |  | N.A. |
| Gargari (2016) |  |  |  |
| Hiraku (2023) |  |  |  |
| Lee (2022) |  |  |  |
| Maneerat (2013) |  |  |  |
| Nakamura (2022) | N.A. | N.A. | N.A. |
| Nyangale (2015) |  |  |  |
| Pagliai (2023) |  |  |  |
| Park (2020) |  |  |  |
| Rahayu (2021) |  |  |  |
| Sato (2022) |  | N.A. |  |
| Senda-Sugimoto (2024) |  |  |  |
| Sierra (2010) |  |  |  |
| Son (2020) |  |  |  |
| Stenman (2016) |  |  |  |
| Swanson (2002) |  |  |  |

|  | no significant difference in SCFA levels between groups |
| --- | --- |
|  | there is a higher SCFA levels in the intervention group (probiotics) |
|  | there is a higher SCFA level in the control group (placebo) |
| N.A. | not applicable* |

*If a study did not investigate (or not in a proper way) a specific outcome, “not applicable” is indicated.

Abbreviations: N.A.: not applicable; SCFA: short-chain fatty acid.

# **Table S4.** Risk of bias assessment for parallel design studies - Assignment to intervention (the 'intention-to-treat' effect).

| **Intention-to-treat_Parallel** |  |  |  |  |  |  |  |  | Low risk |
| --- | --- | --- | --- | --- | --- | --- | --- | --- | --- |
| **Study** | **D1** | **D2** | **D3** | **D4** | **D5** | **Overall** |  |  | Some concerns |
| Chaiyasut et al. |  |  |  |  |  |  |  |  | High risk |
| Park et al. |  |  |  |  |  |  |  |  |  |
| Lee et al. |  |  |  |  |  |  |  | D1 | Randomization process |
| Pagliai et al. |  |  |  |  |  |  |  | D2 | Deviations from the intended interventions |
| Rahayu et al. |  |  |  |  |  |  |  | D3 | Missing outcome data |
| Sierra et al. |  |  |  |  |  |  |  | D4 | Measurement of the outcome |
| Sato et al. |  |  |  |  |  |  |  | D5 | Selection of the reported result |

# **Table S5.** Risk of bias assessment for parallel design studies - Adhering to intervention (the 'per-protocol' effect).

| **Per-protocol_Parallel** |  |  |  |  |  |  |  |  | Low risk |
| --- | --- | --- | --- | --- | --- | --- | --- | --- | --- |
| **Study** | **D1** | **D2** | **D3** | **D4** | **D5** | **Overall** |  |  | Some concerns |
| Alba et al. |  |  |  |  |  |  |  |  | High risk |
| Bazanella et al. |  |  |  |  |  |  |  |  |  |
| Freedman et al. |  |  |  |  |  |  |  |  |  |
| Hiraku et al. |  |  |  |  |  |  |  | D1 | Randomization process |
| Senda-Sugimoto et al. |  |  |  |  |  |  |  | D2 | Deviations from the intended interventions |
| Son et al. |  |  |  |  |  |  |  | D3 | Missing outcome data |
| Stenman et al. |  |  |  |  |  |  |  | D4 | Measurement of the outcome |
| Swanson et al. |  |  |  |  |  |  |  | D5 | Selection of the reported result |

# **Table S6.** Risk of bias assessment for crossover design studies - Adhering to intervention (the 'per-protocol' effect).

| **Per-protocol Crossover** |  |  |  |  |  |  |  |  |  | Low risk |
| --- | --- | --- | --- | --- | --- | --- | --- | --- | --- | --- |
| **Study** | **D1** | **DS** | **D2** | **D3** | **D4** | **D5** | **Overall** |  |  | Some concerns |
| Boesmans et al. |  |  |  |  |  |  |  |  |  | High risk |
| Childs et al. |  |  |  |  |  |  |  |  |  |  |
| Ferrario et al. |  |  |  |  |  |  |  |  | D1 | Randomization process |
| Gargari et al. |  |  |  |  |  |  |  |  | DS | Bias arising from period and carryover effects |
| Maneerat et al. |  |  |  |  |  |  |  |  | D2 | Deviations from the intended interventions |
| Nyangale et al. |  |  |  |  |  |  |  |  | D3 | Missing outcome data |
|  |  |  |  |  |  |  |  |  | D4 | Measurement of the outcome |
|  |  |  |  |  |  |  |  |  | D5 | Selection of the reported result |

# **Table S7.** GRADE assessment for the meta-analyses of faecal butyrate, acetate, and propionate concentrations.

| **Certainty assessment** | | | | | | | **№ of patients** | | **Effect** | | **Certainty** | **Importance** |
| --- | --- | --- | --- | --- | --- | --- | --- | --- | --- | --- | --- | --- |
| **№ of studies** | **Study design** | **Risk of bias** | **Inconsistency** | **Indirectness** | **Imprecision** | **Other considerations** | **probiotics** | **placebo** | **Relative (95% CI)** | **Absolute (95% CI)** |  |  |
| **Butyrate (assessed with: different in vitro methods)** | | | | | | | | | | | | |
| 13 | randomized trials | serious | not serious | serious | not serious | none | 338 | 347 | - | SMD **0.04 SD higher** (0.18 lower to 0.26 higher) | ⨁⨁◯◯ Low | IMPORTANT |
| **Acetate (follow-up: range 21 days to 1 years; assessed with: different in vitro methods)** | | | | | | | | | | | | |
| 11 | randomized trials | serious | not serious | serious | not serious | none | 218 | 219 | - | SMD **0.04 SD higher** (0.15 lower to 0.23 higher) | ⨁⨁◯◯ Low | IMPORTANT |
| **Propionate (follow-up: range 21 days to 6 months; assessed with: different in vitro methods)** | | | | | | | | | | | | |
| 12 | randomized trials | serious | not serious | serious | not serious | none | 315 | 327 | - | SMD **0.08 SD higher** (0.07 lower to 0.24 higher) | ⨁⨁◯◯ Low | IMPORTANT |

CI: confidence interval; SMD: standardized mean difference
