# Supplementary material for: Effects of probiotic supplementation on faecal short-chain fatty acid concentrations in healthy individuals: a systematic review and meta-analysis of randomized controlled trials
Source: Nutr J. 2026 Jul 13;25:106. doi: 10.1186/s12937-026-01364-0 (PMC13644228; doi:10.1186/s12937-026-01364-0)
Supplement: Supplementary file 2 [file 12937_2026_1364_MOESM2_ESM.docx]

**Supplementary Material 2 0 Figures**

**Contents**

[**Fig. S1.** Additional sensitivity analysis for the baseline values of faecal butyrate concentration.	2](#_Toc203166584)

[**Fig. S2.** Additional sensitivity analysis for the subgroups based on study design for faecal butyrate concentration.	3](#_Toc203166585)

[**Fig. S3.** Additional sensitivity analysis for the subgroups based on risk of bias assessment for faecal butyrate concentration.	4](#_Toc203166586)

[**Fig. S4.** Additional sensitivity analysis for the baseline values of faecal acetate concentration.	5](#_Toc203166587)

[**Fig. S5.** Additional sensitivity analysis for the subgroups based on study design for faecal acetate concentration.	6](#_Toc203166588)

[**Fig. S6.** Additional sensitivity analysis for the subgroups based on risk of bias assessment for faecal acetate concentration.	7](#_Toc203166589)

[**Fig. S7.** Additional sensitivity analysis for the baseline values of faecal propionate concentration.	8](#_Toc203166590)

[**Fig. S8.** Additional sensitivity analysis for the subgroups based on study design for faecal propionate concentration.	9](#_Toc203166591)

[**Fig. S9.** Additional sensitivity analysis for the subgroups based on risk of bias assessment for faecal propionate concentration.	10](#_Toc203166592)

[**Fig. S10.** Funnel plot representing publication bias for faecal butyrate concentration.	11](#_Toc203166593)

[**Fig. S11.** Additional leave-one-out analysis for faecal butyrate concentration.	12](#_Toc203166594)

[**Fig. S12.** Funnel plot representing publication bias for faecal acetate concentration.	13](#_Toc203166595)

[**Fig. S13.** Additional leave-one-out analysis for faecal acetate concentration.	14](#_Toc203166596)

[**Fig. S14.** Funnel plot representing publication bias for faecal propionate concentration.	15](#_Toc203166597)

[**Fig. S15.** Additional leave-one-out analysis for faecal propionate concentration.	16](#_Toc203166598)

[**Fig. S16.** Risk of bias assessment for parallel design studies - Assignment to intervention (the 'intention-to-treat' effect).	17](#_Toc203166599)

[**Fig. S17.** Risk of bias assessment for parallel design studies - Adhering to intervention (the 'per-protocol' effect).	18](#_Toc203166600)

[**Fig. S18.** Risk of bias assessment for crossover design studies - Adhering to intervention (the 'per-protocol' effect).	19](#_Toc203166601)

# **Fig. S1.** Additional sensitivity analysis for the baseline values of faecal butyrate concentration.

Abbreviations: SMD: standardized mean difference; SD: standard deviation; CI: confidence interval.

# **Fig. S2.** Additional sensitivity analysis for the subgroups based on study design for faecal butyrate concentration.

Abbreviations: SMD: standardized mean difference; SD: standard deviation; CI: confidence interval.

# **Fig. S3.** Additional sensitivity analysis for the subgroups based on risk of bias assessment for faecal butyrate concentration.

Abbreviations: SMD: standardized mean difference; SD: standard deviation; CI: confidence interval.

# **Fig. S4.** Additional sensitivity analysis for the baseline values of faecal acetate concentration.

Abbreviations: SMD: standardized mean difference; SD: standard deviation; CI: confidence interval.

# **Fig. S5.** Additional sensitivity analysis for the subgroups based on study design for faecal acetate concentration.

Abbreviations: SMD: standardized mean difference; SD: standard deviation; CI: confidence interval.

# **Fig. S6.** Additional sensitivity analysis for the subgroups based on risk of bias assessment for faecal acetate concentration.

Abbreviations: SMD: standardized mean difference; SD: standard deviation; CI: confidence interval.

# **Fig. S7.** Additional sensitivity analysis for the baseline values of faecal propionate concentration.

Abbreviations: SMD: standardized mean difference; SD: standard deviation; CI: confidence interval.

# **Fig. S8.** Additional sensitivity analysis for the subgroups based on study design for faecal propionate concentration.

Abbreviations: SMD: standardized mean difference; SD: standard deviation; CI: confidence interval.

# **Fig. S9.** Additional sensitivity analysis for the subgroups based on risk of bias assessment for faecal propionate concentration.

Abbreviations: SMD: standardized mean difference; SD: standard deviation; CI: confidence interval.

# **Fig. S10.** Funnel plot representing publication bias for faecal butyrate concentration.

The points on the funnel plots for assessing publication bias represent the different studies. It shows the residuals on the x-axis against their corresponding standard errors. Here we could assess the small study bias: at the bottom of the funnel if the studies are distributed not symmetrically and out of the funnel, it indicates potential publication bias.

# **Fig. S11.** Additional leave-one-out analysis for faecal butyrate concentration.

Cases which are considered as possible influential with respect to any of the shown measures are marked with “*” at column “*Influential*”. Note that the chosen cut-offs are (somewhat) arbitrary based on *dmetar* package. “*Effect size*”: the pooled effect size without the given study.

“*95% CI*”: the 95% confidence interval of the pooled effect size without the given study. “*I^2^*”: the Higgins&Thomson I^2^ heterogeneity value without the given study. “*Std residual*”: the studentized residuals. It shows the deleted residual divided by its estimated standard deviation.

“*Dffits*”: the difference in fits. It quantifies the number of standard deviations that the fitted value changes without the given study. (Typical threshold is 3∗ √(p/(k-p)), where *p* is the number of model coefficients and *k* is the number of cases). “*Cook’s dist.*”: Cook’s distance. It depends on both the residual and leverage of the omitted study. (Typical threshold value is 2). “*Covariance ratio*”: the covariance ratio. It shows the change in the determinant of the covariance matrix of the effect size. (Typical threshold value is 1). “*Hat value*”: the value of the hat matrix without the given study. (Typical threshold is 3∗p/k)

Abbreviations: SMD: standardized mean difference; CI: confidence interval.

# **Fig. S12.** Funnel plot representing publication bias for faecal acetate concentration.

The points on the funnel plots for assessing publication bias represent the different studies. It shows the residuals on the x-axis against their corresponding standard errors. Here we could assess the small study bias: at the bottom of the funnel if the studies are distributed not symmetrically and out of the funnel, it indicates potential publication bias.

# **Fig. S13.** Additional leave-one-out analysis for faecal acetate concentration.

Cases which are considered as possible influential with respect to any of the shown measures are marked with “*” at column “*Influential*”. Note that the chosen cut-offs are (somewhat) arbitrary based on *dmetar* package. “*Effect size*”: the pooled effect size without the given study.

“*95% CI*”: the 95% confidence interval of the pooled effect size without the given study. “*I^2^*”: the Higgins&Thomson I^2^ heterogeneity value without the given study. “*Std residual*”: the studentized residuals. It shows the deleted residual divided by its estimated standard deviation.

“*Dffits*”: the difference in fits. It quantifies the number of standard deviations that the fitted value changes without the given study. (Typical threshold is 3∗ √(p/(k-p)), where *p* is the number of model coefficients and *k* is the number of cases). “*Cook’s dist.*”: Cook’s distance. It depends on both the residual and leverage of the omitted study. (Typical threshold value is 2). “*Covariance ratio*”: the covariance ratio. It shows the change in the determinant of the covariance matrix of the effect size. (Typical threshold value is 1). “*Hat value*”: the value of the hat matrix without the given study. (Typical threshold is 3∗p/k)

Abbreviations: SMD: standardized mean difference; CI: confidence interval.

# **Fig. S14.** Funnel plot representing publication bias for faecal propionate concentration.

The points on the funnel plots for assessing publication bias represent the different studies. It shows the residuals on the x-axis against their corresponding standard errors. Here we could assess the small study bias: at the bottom of the funnel if the studies are distributed not symmetrically and out of the funnel, it indicates potential publication bias.

# **Fig. S15.** Additional leave-one-out analysis for faecal propionate concentration.

Cases which are considered as possible influential with respect to any of the shown measures are marked with “*” at column “*Influential*”. Note that the chosen cut-offs are (somewhat) arbitrary based on *dmetar* package. “*Effect size*”: the pooled effect size without the given study.

“*95% CI*”: the 95% confidence interval of the pooled effect size without the given study. “*I^2^*”: the Higgins&Thomson I^2^ heterogeneity value without the given study. “*Std residual*”: the studentized residuals. It shows the deleted residual divided by its estimated standard deviation.

“*Dffits*”: the difference in fits. It quantifies the number of standard deviations that the fitted value changes without the given study. (Typical threshold is 3∗ √(p/(k-p)), where *p* is the number of model coefficients and *k* is the number of cases). “*Cook’s dist.*”: Cook’s distance. It depends on both the residual and leverage of the omitted study. (Typical threshold value is 2). “*Covariance ratio*”: the covariance ratio. It shows the change in the determinant of the covariance matrix of the effect size. (Typical threshold value is 1). “*Hat value*”: the value of the hat matrix without the given study. (Typical threshold is 3∗p/k)

Abbreviations: SMD: standardized mean difference; CI: confidence interval.

# **Fig. S16.** Risk of bias assessment for parallel design studies - Assignment to intervention (the 'intention-to-treat' effect).

# **Fig. S17.** Risk of bias assessment for parallel design studies - Adhering to intervention (the 'per-protocol' effect).

# **Fig. S18.** Risk of bias assessment for crossover design studies - Adhering to intervention (the 'per-protocol' effect).
